# Decreased TSPAN1 promotes prostate cancer progression and is a marker for early biochemical recurrence after radical prostatectomy

**DOI:** 10.18632/oncotarget.11448

**Published:** 2016-08-20

**Authors:** Fan Xu, Yujing Gao, Yanqing Wang, Jiahua Pan, Jianjun Sha, Xiaoguang Shao, Xunlei Kang, Jun Qin, M. James You, Yiran Huang, Baijun Dong, Wei Xue

**Affiliations:** ^1^ Department of Urology, RenJi Hospital, School of Medicine, Shanghai Jiaotong University, Shanghai, China; ^2^ Key Laboratory of Fertility Preservation and Maintenance of Ministry of Education, Department of Biochemistry and Molecular Biology, Ningxia Medical University, Yinchuan, Ningxia, China; ^3^ Division of Oncology, Department of Medicine, University of Missouri School of Medicine, Columbia, MO, USA; ^4^ Institute of Health Sciences, Shanghai Institutes for Biological Sciences, Chinese Academy of Sciences, Shanghai, China; ^5^ Department of Molecular and Cellular Biology, Baylor College of Medicine, Houston, TX, USA; ^6^ Program of Genes and Development, The University of Texas Graduate School of Biomedical Sciences, Houston, TX, USA; ^7^ Department of Hematopathology, Division of Pathology and Laboratory Medicine, The University of Texas MD Anderson Cancer Center, Houston, TX, USA

**Keywords:** prostate cancer, TSPAN1, progression, prognosis, biochemical recurrence

## Abstract

Patients with prostate cancer (PCa) have a variable prognosis. It is challenging to recognize the progressive disease. In this study, we focused on TSPAN1, a new member of the tetraspanin family. Its expression was decreased in progressive PCa and was an independent prognosis factor of biochemical recurrence after radical prostatectomy. *In vitro*, knockdown and overexpression of TSPAN1 in PCa cell lines showed that TSPAN1 could inhibit cell proliferation and migration. TSPAN1 was positive related to PTEN in both clinical specimen and mouse models. The combination of these two markers could increase their prognosis value especially in low risk patients. In vitro TSPAN1 knockdown resulted in increased Akt phosphorylation and caused evident cell cycle transition from G1 to S phase. Our data suggests that TSPAN1 is a valuable marker to recognize more progressive PCa.

## INTRODUCTION

Prostate cancer is the most diagnosed and the second death leading cancer in the United States [1]. Radical prostatectomy (RP) is a standard method to treat localized prostate cancer. Biochemical relapse is a sensitive marker to predict tumor recurrence after the operation, including local recurrence and metastasis [2]. Identifying risk factors for metastasis progression in patients with biochemical recurrence is crucial. Patients with low risk of metastasis are response well to locally salvage therapy [3]. However, clinical methods to detect potential metastasis progression cancer still remain some problems [4]. It is reported that many patients within the low-risk subgroup have an excellent outcome even without any salvage treatment after biochemical recurrence [5]. This phenomenon indicates variation among this group of patients, which may make treatment in dilemma. It suggests that the molecular differences should be taken into consideration [6].

Tetraspanins are a heterogeneous group of four transmembrane proteins, which are expressed across evolution from sponges to mammals [7]. Studies have established that they can affect tumor metastasis by forming tetraspanin-enriched membrane micro-domains (TEMs) with other tetraspanins and with a variety of trans-membrane and cytosolic proteins through different mechanisms. For example, CD82 is recognized as a typical metastasis suppressor. It can interact with integrin and then form a protein complex with c-Met. Through the recruitment of regulation cofactors, CD82 influences the protein complex function, which results in promoting matrix adhesion and inducing senescence [8–9]. On the other hand, CD151 and tetraspanin8 are shown to promote metastasis by supporting cell motility [10–12]. The controversial role of tetraspanins in tumor progression may be due to their function of serving as a scaffold to interact with various proteins.

TSPAN1 is a novel member of the tetraspanin family [13], and its role in tumor progression is not entirely understood. Studies in gastric, colonic, and cervical cancer have shown that TSPAN1 can promote tumor cell proliferation and invasion *in vitro* and its expression is elevated in these primary tumor tissues of human [14–16]. However, in breast cancer it is reported that although TSPAN1 gene is amplified in most non-metastasis primary lesions, it is more likely to be obliterated in metastasis matched primary lesions [17]. Thus, TSPAN1 has a clinical potential in distinguishing more progressive patients after operation or biopsy. To our knowledge, TSPAN1 expression panel in prostate cancer was still unreported, and all of the researches above didn't show possible mechanisms of TSPAN1. Therefore, we decided to investigate clinical significance of TSPAN1 in our postoperative prostate cancer cohort and attempted to explore its function.

## RESULTS

### TSPAN1 expression was decreased in metastasis prostate cancer lesions

**Figure 1:**
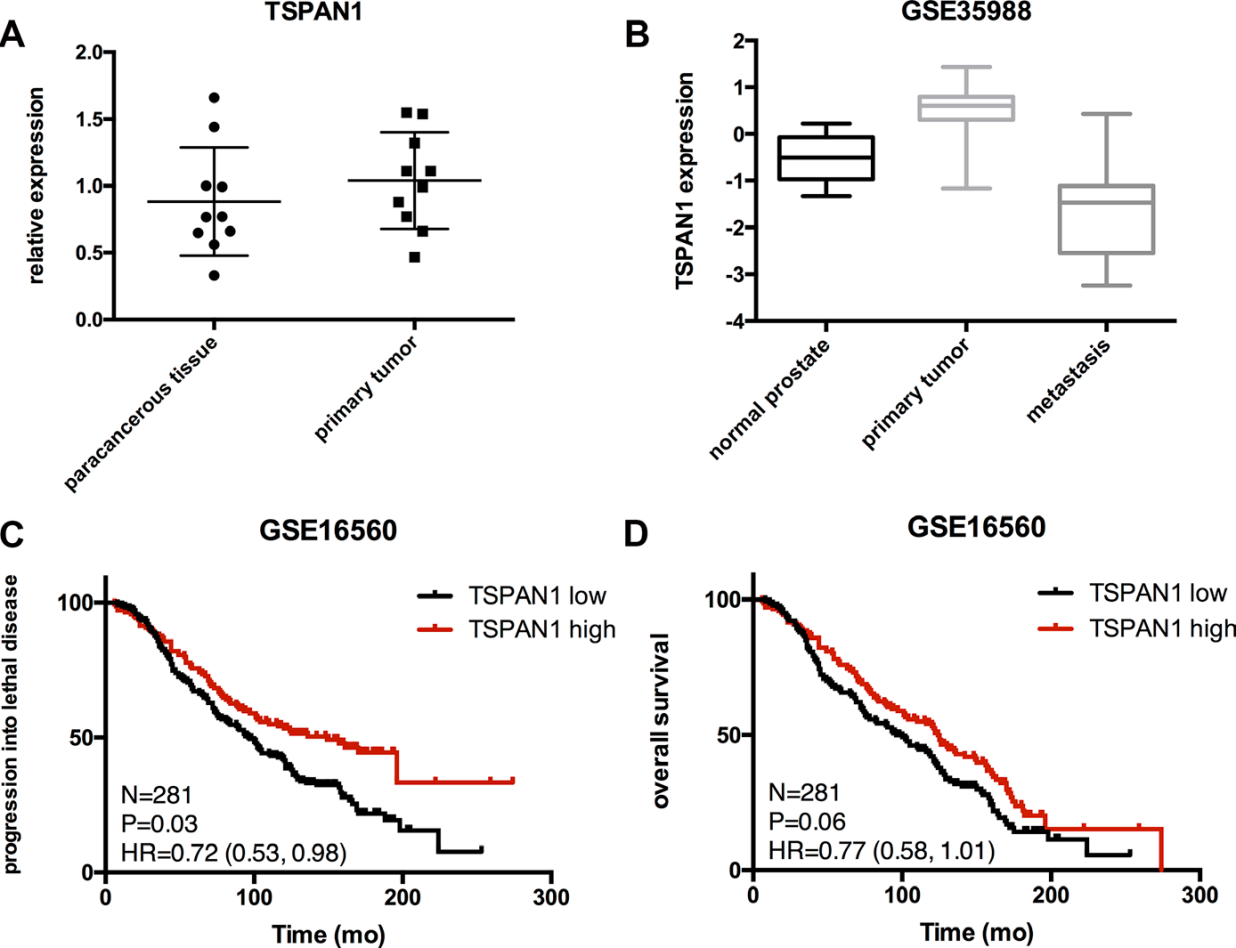
Expression of TSPAN1 in samples and published databases (**A**) TSPAN1 mRNA level in prostate tumors and corresponding paracancerous tissues were obtained using quantitative real-time PCR. TSPAN1 mRNA expression levels were normalized to GAPDH mRNA expression. There were no significant differences between the two groups (*n* = 10, paired *t* test *P* = 0.2971). (**B**) Expression of TSPAN1 was significantly decreased in metastasis lesions compared to primary cancer and normal tissues (vs. normal prostate *P* = 0.0004, vs. primary tumor *P* < 0.0001). (**C** and **D**) In a Swedish watchful waiting cohort, patients with low expression of TSPAN1 display a significant shorter time of progressing into lethal disease (metastasis or disease specific death, *P* = 0.036) and a tendency of shorter overall survival but not significant (*P* = 0.06). Low expression was defined by lower than average expression level of this cohort.

TSPAN1 expression was tested by qPCR in our frozen human prostate cancer samples and paired paracancerous tissues. The result analyzed by paired *t-test* did not show any differences, *P* = 0.2977 (Figure 1A). Expression of TSPAN1 in prostate cancer patients was also explored in published datasets. It was found to be significantly decreased in metastasis lesions vs. normal prostate *P* = 0.0004; vs. primary tumor *P* < 0.0001 (Figure 1B, from GSE35988 dataset). The expression pattern of TSPAN1 in prostate cancer was consistent with that from the breast cancer [17]. Analysis of data from GSE16560, derived from a prominent Swedish watchful waiting cohort [18], the decrease of TSPAN1 expression was significantly related to the early occurrence of disease progression (Figure 1C). Although its effect on overall survival was not significant (Figure 1D), TSPAN1 could be a marker that distinguishes more progressive prostate cancer from the indolent.

### TSPAN1 expression was an independent prognosis factor of biochemical recurrence after RP

**Figure 2:**
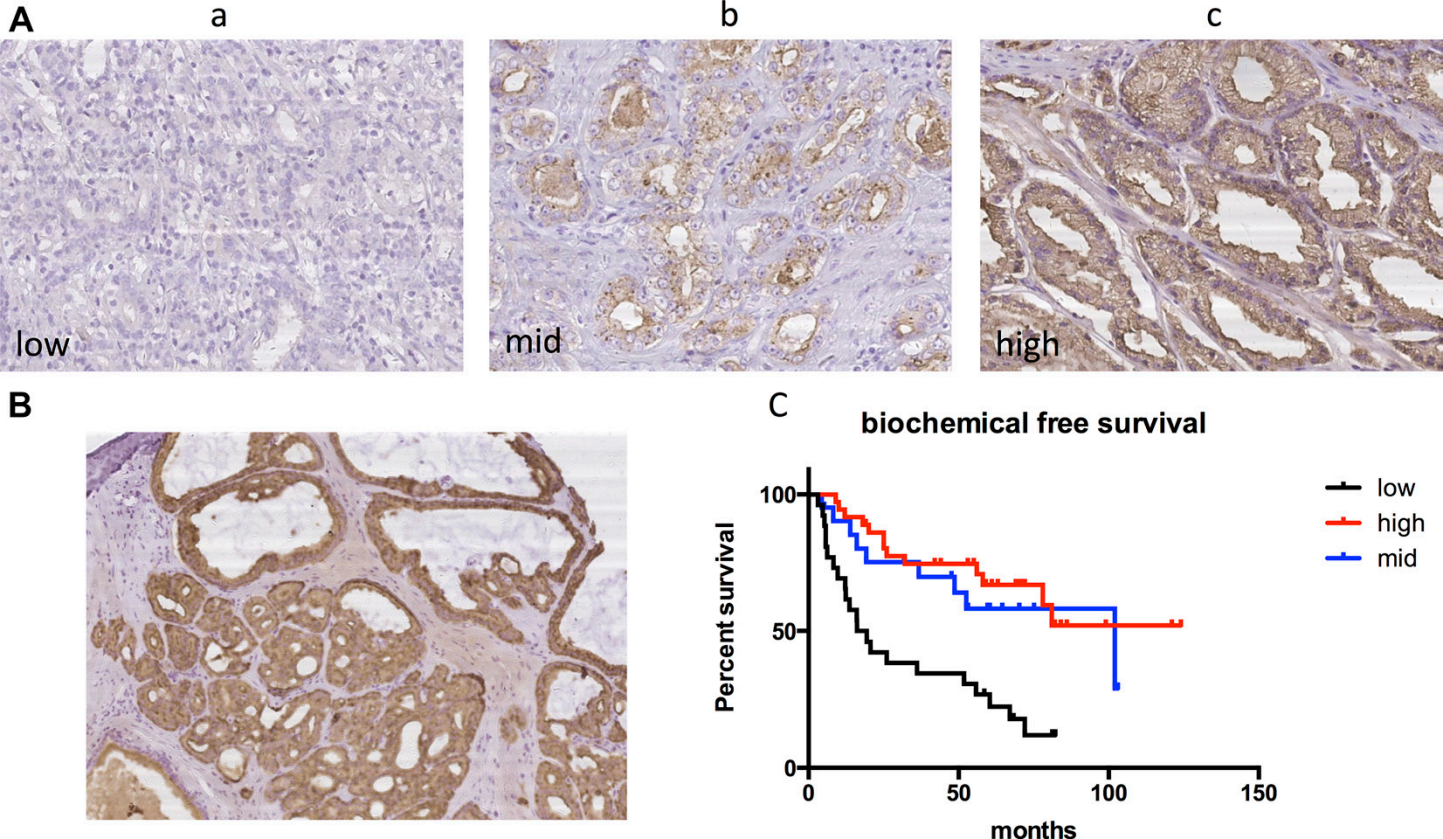
TSPAN1 protein expression in our radical prostatectomy cohort (**A**) Different expression levels of TSPAN1 in postoperative tissue samples. a) low expression b) mid expression c) high expression (20 × magnification). (**B**) TSPAN1 protein expression had no significant differences between tumor (left inferior) and paracancerous tissues (right superior) (10 × magnification). (**C**) Kaplan-Meier method and log-rank test were used to evaluate biochemical recurrence free survival and compare the differences. Low expression level of TSPAN1 predicted early biochemical recurrence after radical prostatectomy, high vs. low expression *P* < 0.0001 HR = 0.262(0.102,0.428), mid vs. low expression *P* = 0.007 HR = 0.373(0.183,0.747).

We further assessed TSPAN1 protein expression in our Asian radical prostatectomy cohort and divided it into three levels according to the criteria described in the method (Figure 2A). The protein expression had no significant differences between tumor and paracancerous tissues (Figure 2B). The tumor characteristics and clinical features of 118 patients were summarized in Table 1, and TSPAN1 expression was not significantly correlated with other clinical characteristics (Table 2). However, the low expression level was significantly related to the early biochemical recurrence after radical prostatectomy (Figure 2C), high vs. low expression *P* < 0.0001 HR = 0.262 (0.102,0.428), mid vs. low expression *P* = 0.007 HR = 0.373 (0.183,0.747). According to log rank test, high baseline PSA, high Gleason score were also significantly related to early biochemical recurrence. Multivariate analysis with cox regression showed that TSPAN1 expression level was an independent prognosis factor of biochemical recurrence after RP and was superior to the other two variables, *P* = 0.002 HR = 0.669 (0.518,0.865) (Table 3). TSPAN1 expression was decreased in more progressive prostate tumors, and it could be used to predict the early biochemical recurrence after radical prostatectomy.

**Table 1:** Clinical characteristic of patients in the study

Variables	All patients
Numbers, *n* (%)	118 (100)
Age at diagnosis, yr.	66 (61–71)
Year of surgery	2009 (2007–2010)
No. of biochemical recurrence, *n* (%)	47 (39.8)
Follow-up time of censored patients, yr.	6.4 (5.6–7.4)
Preoperative PSA, ng/mL	16.0 (10.4–31.6)
Pathologic Gleason score, *n* (%)	
≤ 6	29 (24.6)
7	53 (44.9)
8	21 (17.8)
≥ 9	15 (12.7)
Adverse pathologic events, *n* (%)	
Extra-prostatic extension	26 (22.0)
Seminal vesicle invasion	13 (11.0)
Lymph node invasion	4 (3.4)
Positive surgical margins	6 (5.1)
TSPAN1 expression score, *n* (%)	
Low	26 (22.0)
Moderate	24 (20.3)
High	58 (49.2)

**Table 2:** Correlation between TSPAN1 expression and multiple clinicopathological characteristics

Variables	TSPAN1 expression			*P* value
	Low	Mid	High	
Age at diagnosis, yr				0.124
< 66	10 (18.2%)	10 (18.2%)	35 (63.6%)	
≥ 66	16 (30.8%)	13 (25.0%)	23 (44.2%)	
Preoperative PSA, ng/mL				0.279
< 16.0	9 (18.8%)	13 (27.1%)	26 (54.2%)	
≥ 16.0	16 (28.1%)	9 (15.8%)	32 (56.1%)	
Adverse pathologic events				0.468
Without APE	18 (21.7%)	18 (21.7%)	47 (56.6%)	
With APE	8 (32.0%)	6 (24.0%)	11 (44.0%)	
Pathologic Gleason score				0.818
≤ 6	5 (22.7%)	4 (18.2%)	13 (59.1%)	
7	11 (21.6%)	13 (25.5%)	27 (52.9%)	
≥ 8	10 (31.3%)	7 (21.9%)	15 (46.9%)	

**Table 3:** TSPAN1 is an independent prognosis factor of biochemical recurrence after RP*

Variables	Biochemical recurrence			Log rank test		Cox regression analysis	
	Groups	No.	Median survival (mo.)	*P* value	HR (95% CL)	*P* value	HR (95% CL)
Total		47	72				
Age at diagnosis	< 66	20	72	0.875	1.047 (0.586, 1.872)		
	≥ 66	27	60				
Year of surgery	Before 2009	28	36	0.103	0.635 (0.344, 1.080)		
	After 2009	19	72				
Preoperative PSA, ng/mL	< 16.0	17	81	0.004	2.339 (1.320, 4.191)	0.123	1.679 (0.869, 3.244)
	≥ 16.0	30	26				
Adverse pathologic events	Without APE**	37	78	0.163	1.629 (0.793, 4.075)		
	With APE	10	58				
Pathologic Gleason score	≤ 6	11	102		1	0.360	1.224 (0.794, 1.887)
	7	22	72	0.169	1.649 (0.817, 3.218)		
	≥ 8	13	26	0.021	2.438 (1.190, 6.558)		
TSPAN1 expression score	Low	22	18		1	0.002	0.669 (0.518, 0.865)
	Moderate	9	102	0.007	0.373 (0.183, 0.747)		
	High	13	Undefined	< 0.0001	0.262 (0.102, 0.428)		

* RP: radical prostatectomy;

** APE: adverse pathologic events, including extra-prostatic extension, seminal vesicle invasion, lymph node invasion, and positive surgical margins.

### TSPAN1 suppressed proliferation and migration of prostate cancer cells *in vitro*

**Figure 3:**
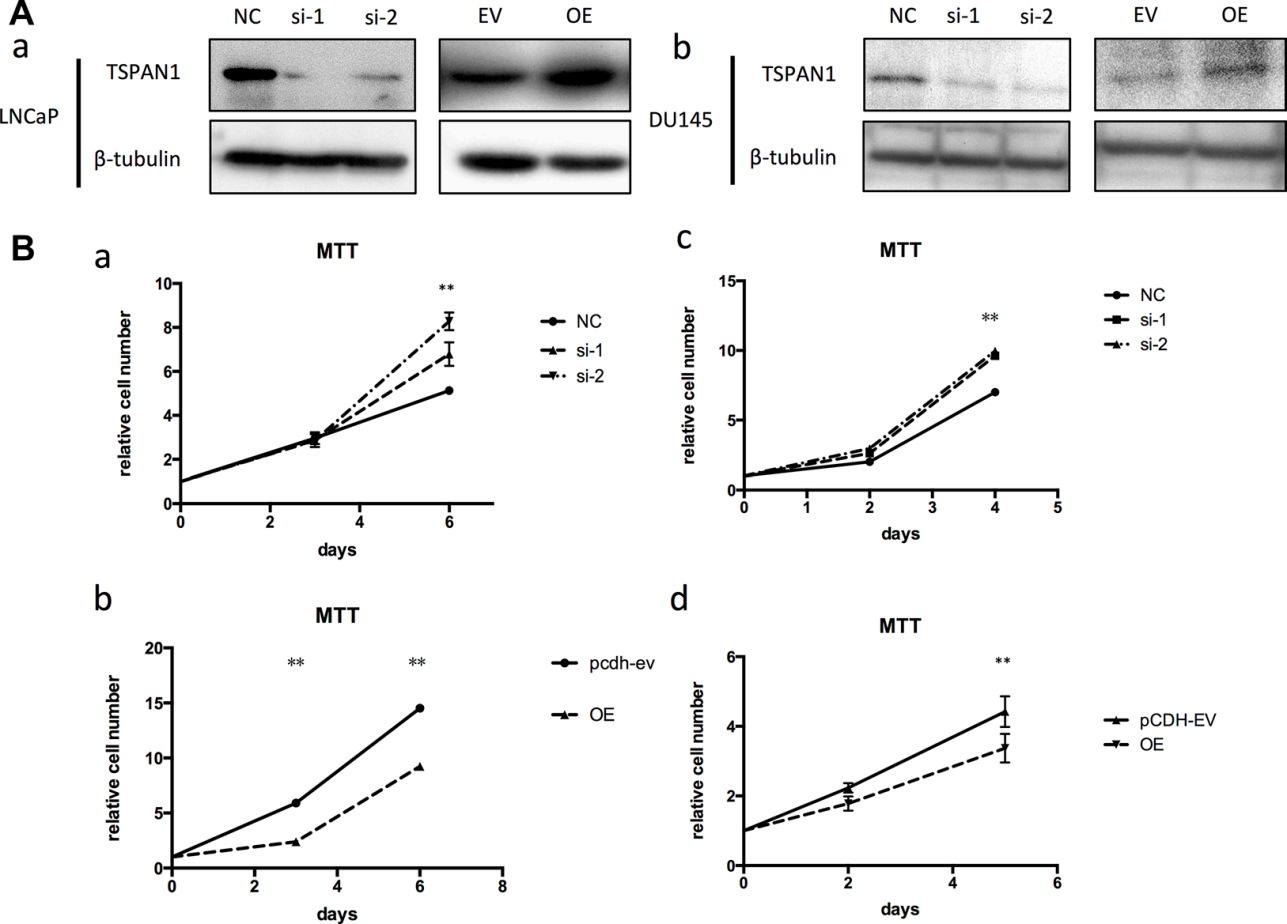
TSPAN1 affected proliferation of prostate cancer cells *in vitro* (**A**) Knockdown and overexpression of TSPAN1 in LNCaP and DU145 cell lines analyzed by western blot. β-tubulin was used as a loading control for western blot assays. (**B**) MTT assay was performed to evaluate the TSPAN1 on the proliferation of LNCaP and DU145 at indicated time points. a) TSPAN1 knockdown promoted cell proliferation in LNCaP; b) TSPAN1 overexpression inhibited cell proliferation in LNCaP; c) TSPAN1 knockdown promoted cell proliferation in DU145; d) TSPAN1 overexpression inhibited cell proliferation in DU145.

**Figure 4:**
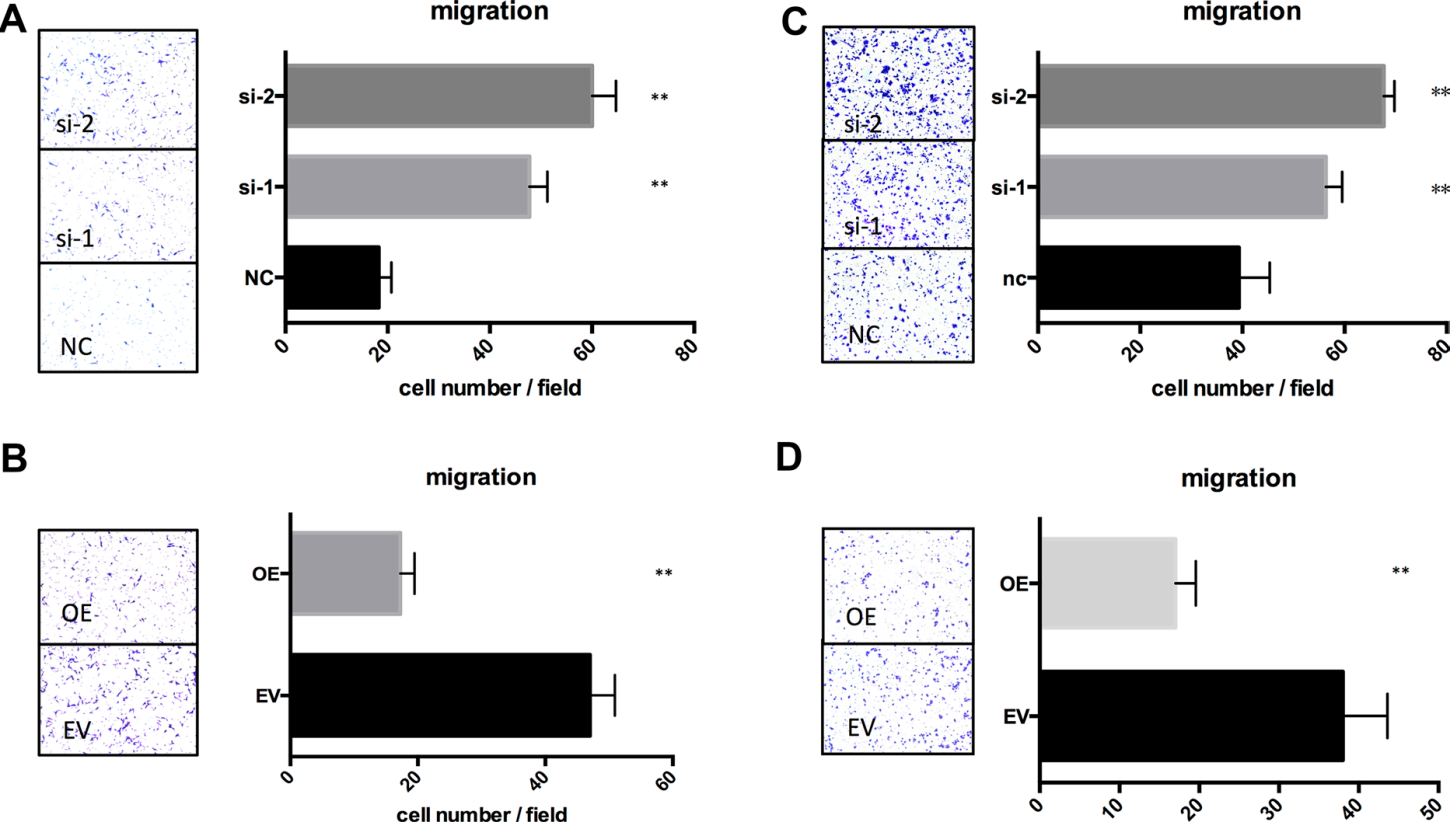
TSPAN1 affected migration of prostate cancer cells *in vitro* Transwell assay was performed to evaluate the TSPAN1 on the migration of LNCaP and DU145. LNCaP was fixed in 20% methanol and stained by 0.1% crystal violet after 42 h; DU145 was fixed and stained after 12 h. (**A**) TSPAN1 knockdown promoted cell migration in LNCaP; (**B**) TSPAN1 overexpression inhibited cell migration in LNCaP; (**C**) TSPAN1 knockdown promoted cell migration in DU145; (**D**) TSPAN1 overexpression inhibited cell migration in DU145.

**Figure 5:**
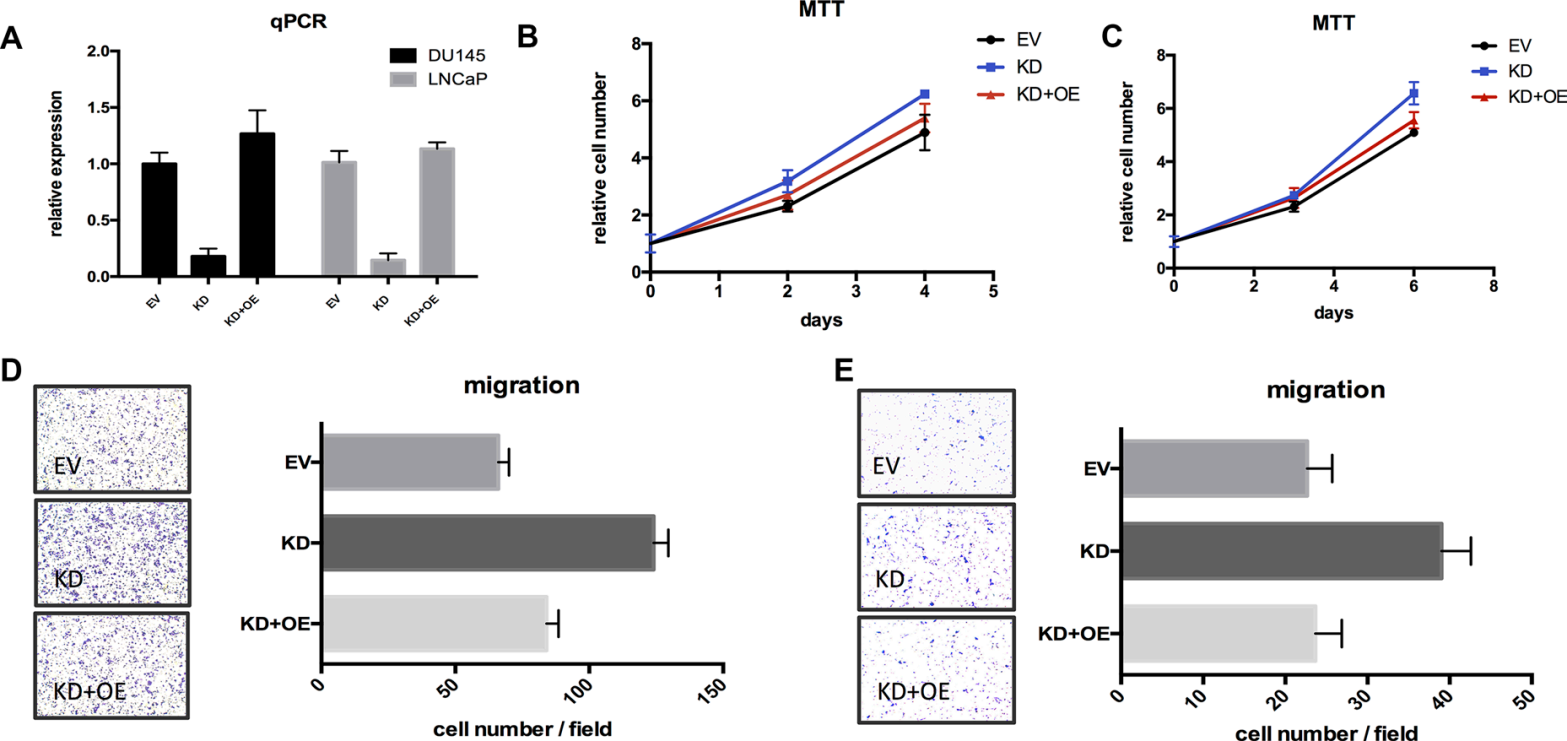
Restoring TSPAN1 in knockdown cells could reverse the aggressive phenotype of proliferation and migration (**A**) TSPAN1 restoring was checked by qPCR test. (**B**) MTT essay of TSPAN1 restoring in DU145 cells. On the 4th day: KD vs. EV, P= 0.0215; KD+OE vs. EV, P= 0.3328. (**C**) MTT essay of TSPAN1 restoring in LNCaP cells. On the 6th day: KD vs. EV, *P* = 0.0039; KD+OE vs. EV, *P* = 0.0629. (**D**) Transwell essay of TSPAN1 restoring in DU145 cells. KD vs. EV, *P* < 0.0001; KD+OE vs. EV, *P* = 0.0637. (**E**) Transwell essay of TSPAN1 restoring in LNCaP cells. KD vs. EV, *P* < 0.0001; KD+OE vs. EV, *P* = 0.8190.

To confirm the role of TSPAN1 during prostate cancer development, we altered its expression in two prostate cancer cell lines LNCaP and DU145 (phenotype of manipulating TSPAN1 in PC3 cells was presented in Supplementary Figure S1A–S1C). Knockdown and overexpression of TSPAN1, respectively, were confirmed by western blot analysis (Figure 3A). MTT assay was performed to assess the effects on the prostate cancer cells proliferation. The results demonstrated that the knockdown of TSPAN1 promoted cell proliferation in both LNCaP and DU145 cells while the overexpression exhibited a contrasting effect (Figure 3B). Changes in cell migration were also analyzed by transwell assay. In accordance with proliferation, decreased TSPAN1 promoted cell migration, whereas overexpression inhibited it (Figure 4) in both the cells. Then we restored TSPAN1 expression in the knockdown cells. In both LNCaP and DU145, re-expressing TSPAN1 could reverse the aggressive phenotype of cell proliferation and migration (Figure 5).

### TSPAN1 expression was positive related to PTEN

**Figure 6:**
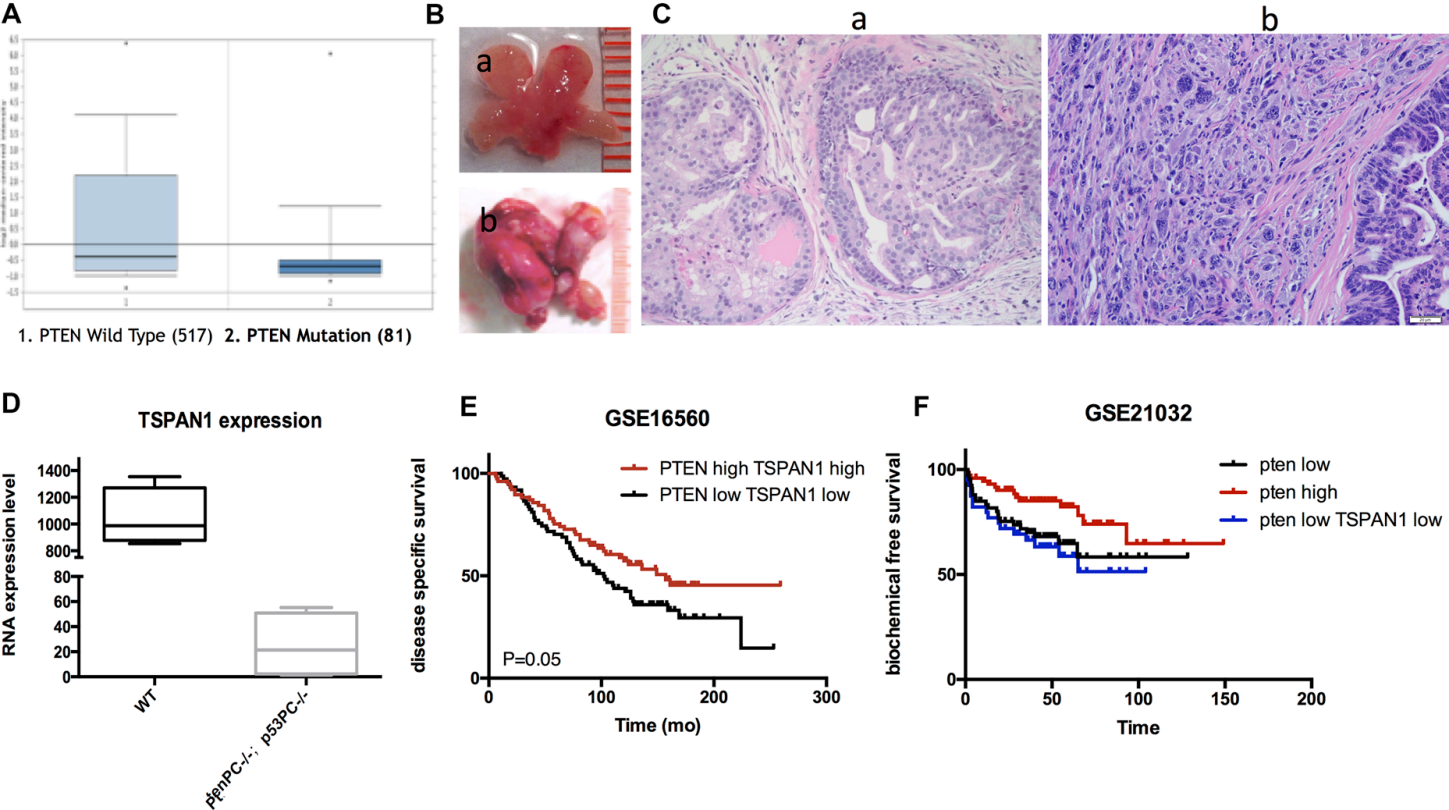
TSPAN1 expression was positive related to PTEN and could increase its prognosis value (**A**) TSPAN1 expression was significant decreased in patients with Pten dysfunction, *P* < 0.01 (data from Oncomine database). (**B**) Prostate samples from a) wild type mouse and b) P53pc−/−Ptenpc−/− mouse at 6 weeks. (**C**) Pathology confirmation of a) normal prostate tissue from wild type mouse and b) invasive prostate cancer tissue from P53pc−/−Ptenpc−/− mouse by HE stain (20 × magnification). (**D**) Sequencing results from P53pc−/−Ptenpc−/− and wild type mouse prostate samples indicated TSPAN1 expression was significantly decreased in P53pc−/− Ptenpc−/− mouse, *P* < 0.01. (**E**) Patients with PTEN TSPAN1 double low expression were significantly progressive than double high expression ones, data from GSE16560, P=0.05. (**F**) Patients with PTEN TSPAN1 double low expression had a tendency of shorter biochemical free survival time than patients only with PTEN low expression, data from GSE21032. PTEN high vs. PTEN low, *P* = 0.020; PTEN high vs. PTEN low TSPAN1 low, *P* =0.007.

We explored the published data from Oncomine and found that TSPAN1 expression was significantly decreased in patients with PTEN dysfunction (Figure 6A). It was reported that PTEN dysfunction was markedly associated with seminal vesicle involvement, extracapsular extension, predicted interval to the development of metastasis, and prostate cancer-specific mortality [19]. As PTEN was a crucial inhibitor of PI3K/Akt pathway, its loss of function could result in a consistent activation of Akt, which is an important mechanism of disease progression in prostate cancer [20]. The sequencing result from our p53^pc−/−^ Pten^pc−/−^ mouse model was in accordance with the published patients' data. Compared to wild type mouse, p53^pc−/−^ Pten^pc−/−^ mouse could form invasive prostate cancer that was invariably lethal by 7 months of age [21]. The sequencing was performed with tumor tissues from p53^pc−/−^ Pten^pc−/−^ mouse and normal prostate from wild-type mouse at 6 weeks (Figure 6B and 6C). TSPAN1 expression was significantly decreased in p53^pc−/−^ Pten^pc−/−^ mouse (Figure 6D) and was confirmed by qPCR test (Supplementary Figure S1D). Although PTEN loss was related to high invasive diseases, we classified patients of GSE16560 database by PTEN expression and found PTEN had limited prognosis value in these low risk patients (data not shown). However, patients with PTEN TSPAN1 double low expression were significantly progressive than double high expression ones (Figure 6E). In the cohort of GSE21032, PTEN expression could predict BFS time (*P* = 0.02), and patients with PTEN TSPAN1 double low expression had a tendency of shorter biochemical free survival time than patients only with PTEN low expression (Figure 6F). Above all, TSPAN1 was positive related to PTEN expression and could add prognosis value of PTEN in predicting disease progression.

### Knockdown of TSPAN1 could activate PI3K/Akt pathway and change cell cycle

**Figure 7:**
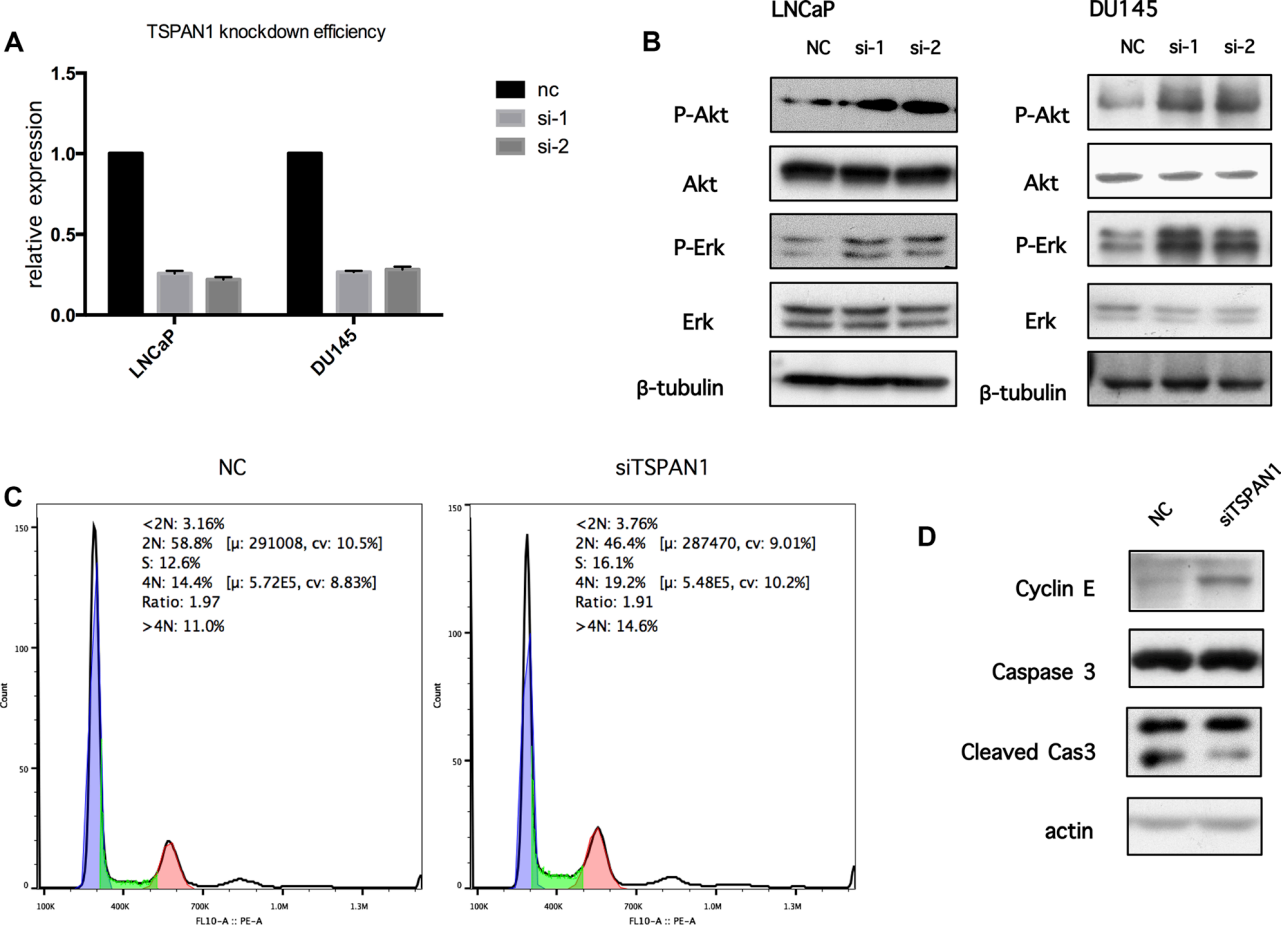
Knockdown of TSPAN1 could activate PI3K/Akt pathway and change cell cycle (**A**) Knockdown efficiency by TSPAN1 siRNA oligo transfection in LNCaP and DU145 was confirmed by real-time RT-PCR on the 6th day after transfection, TSPAN1 mRNA expression was normalized to GAPDH mRNA expression. (**B**) The antibodies against AKT, phospho-AKTS473 (p-AKT), ERK, Phospho-Erk1/2Thr202/Tyr204 (p-ERK) were used to determine the effect of TSPAN1 on the activities of PI3K/Akt and MAPK signaling. TSPAN1 knockdown significantly increased the levels of p-AKT and p-ERK in both LNCaP and DU145 cells. (**C**) The cell cycle phases are analyzed by flow cytometer in LNCaP cells 3 days after transfection (Negtive Control siRNA oligo and TSPAN1 siRNA oilgo). The number of TSPAN1 knockdown cell decreased in G1 (2N) and increased in S and G2 (4N) phases compare to negative control cell. (**D**) CyclinE was increased in TSPAN1 knockdown LNCaP cells compared to negative control cells 3 days after transfection. Caspase 3 and its cleaved forms showed no significant differences.

LNCaP and DU145 cells were transfected with TSPAN1 siRNA to assess the function of PI3K/Akt pathway. Quantitative RT-PCR confirmed the knockdown efficiency (Figure 7A). Although TSPAN1 knockdown did not alter the expression of total Akt, the phosphorylated protein was significantly increased, which indicated the activation of PI3K/Akt pathway. Moreover, we also found that phosphorylated Erk was increased in TSPAN1 knockdown cells (Figure 7B). These results suggested that TSPAN1 might play a major role in cell signal transduction. To further confirm the effect of PI3K/Akt pathway activation caused by the TSPAN1 knockdown, cell cycle, and apoptosis was tested by western blot and flow cytometric analysis in LNCaP cells. Consequent to TSPAN1 knockdown, the proportion of cells in S and G2 phases was noticeably increased (Figure 7C). The Western analysis also showed an up-regulation of cyclinE, which is required for the transition from G1 to S-phase of the cell cycle. There was no significant evidence that TSPAN1 could promote cell apoptosis (Figure 7D).

## DISCUSSION

Tumor recurrence after radical prostatectomy was an adversity in the treatment of prostate cancer. PSA was a sensitive marker of recurrence and should be followed after radical prostatectomy. Early biochemical recurrence was proved to be a predictor of developing metastatic disease [22]. However, a long time follow-up study showed only about 34% of biochemical recurrence patients developed metastatic disease without immediate salvage treatment [23]. This result indicated PSA couldn't distinguish the indolent tumors versus aggressive PCa. The variety of individual patients made the timing of salvage treatment after biochemical recurrence controversial. To solve this problem, some studies explored new molecular markers and showed a large priority of gene tests in distinguishing indolent prostate cancer from progressive ones [24–26]. However, according to our knowledge TSPAN1 wasn't reported before in prostate cancer. Our results suggested that TSPAN1 might be a potential one for further large clinical cohort test. According to our data, decreased TSAPN1 expression was an independent predictor of early biochemical recurrence and was a character of metastasis prostate cancer. Moreover, TSPAN1 overexpression in prostate cancer cells can inhibit proliferation and migration. So we suggested that patients with high expression of TSPAN1 might achieve less benefit from immediate salvage treatment especially when it was thought to be clinical low risk biochemical recurrence.

**Figure 8:**
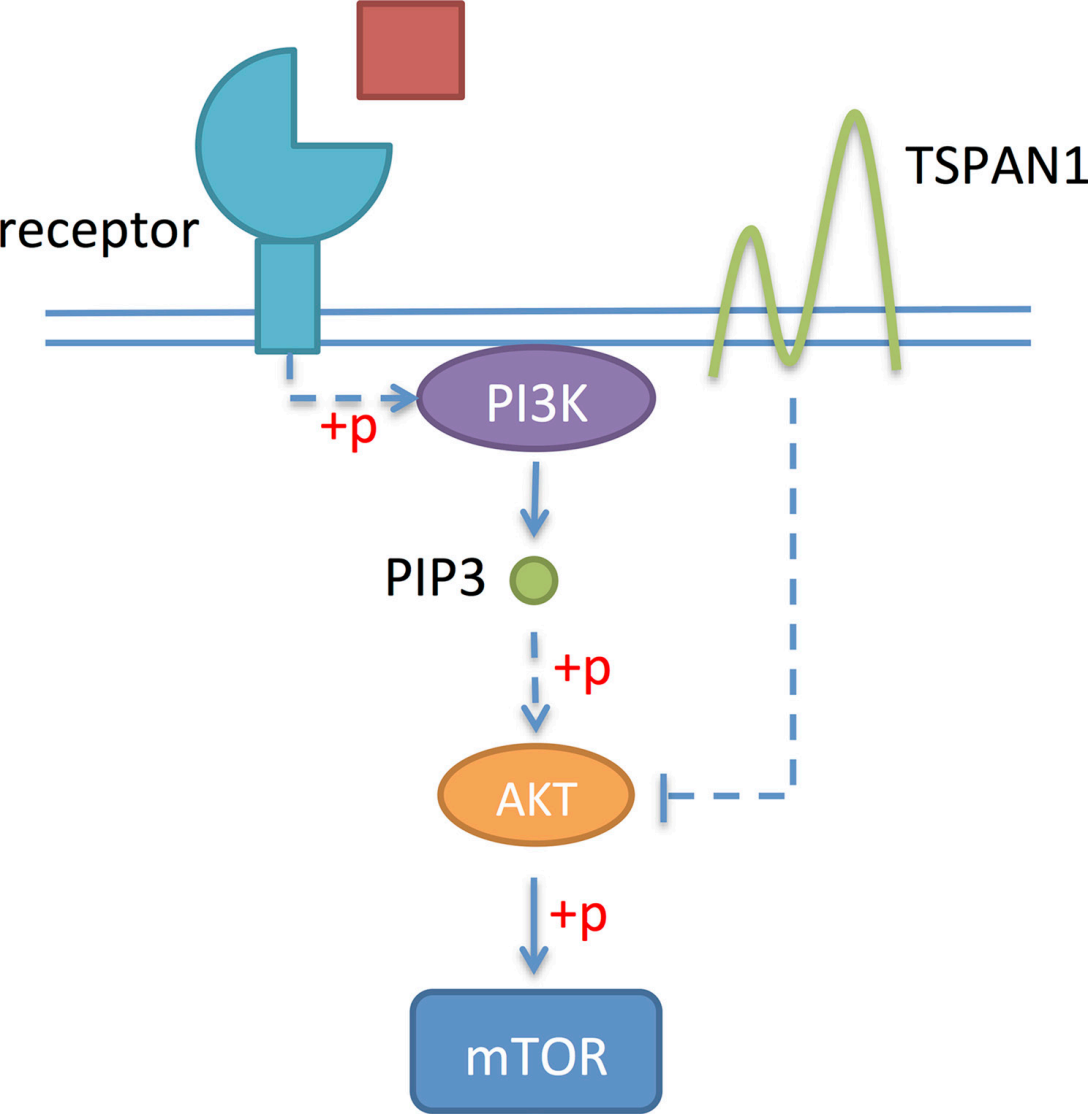
TSPAN1 could suppress the phosphorylation of Akt and inhibit PI3K/Akt pathway

In this study, we first proved that TSPAN1 knockdown increased the phosphorylation level of Akt *in vitro* (Figure 8) and changed cell cycle from phase G1 to S. PI3K/Akt pathway was one of the most prominent alternate pathways in prostate cancer. It may induce disease progression through the activation of downstream growth and survival pathways, which could be reflected as cell cycle changes and apoptosis inhibition [27]. Another method of PI3K/Akt pathway activation in tumors was losing of its suppressor, PTEN. The PTEN gene on chromosome 10q23.3 was the most commonly deleted gene in prostate cancer [28]. Studies have proved that PTEN loss was independently associated with increased risk of lethal progression [29–30]. According to our data, TSPAN1 expression was positive related to PTEN in both patients' specimen and mouse model. What's more, on the basis of PTEN classification, TSPAN1 could enhance the prognosis value of PTEN in prostate cancer patients, especially in clinical low risk groups. Among these patients, PTEN alone didn't have a significant prognosis value.

The study also had limitations. Due to the limitation of follow up time, biochemical recurrence was the only analyzable end point of our cohort right now. Our cohort excluded patients received postoperative adjuvant treatment in order to exclude the interference with BFS from postoperative intervention. As a result, our cohort had a low percentage of high-risk patients, especially the patients with adverse pathology events. We believed the small number might be the reason causing no significant associations between adverse pathology and BCR. And our negative results does not necessarily mean there were no associations for TSPAN1 with APEs, Gleason score, and PSA. In conclusion, decreased expression of TSPAN1 in prostate cancer led to increased cell proliferation and migration, plausibly through the activation of PI3K/Akt pathway. Also, TSPAN1 was an independent prognosis factor of biochemical recurrence after radical prostatectomy and might have practical value especially in clinical low risk patients.

## MATERIALS AND METHODS

### Database analysis

Expression analysis of TSPAN1 in normal prostate, primary tumor, and metastasis tissues was performed on cohorts from microarray databases available on GEO and Oncomine. Gene high or low expression level was divided by median expression value.

### Patients and tissue samples

Clinical parameters of 118 prostate cancer patients who received radical prostatectomy were collected, including age at diagnosis, baseline serum PSA, tumor volume, Gleason score, adverse pathology events (extra-prostatic extension, seminal vesicle invasion, lymph node invasion, and positive surgical margins), and follow-up PSA levels. All of these patients did not receive adjuvant therapy until biochemical recurrence. Biochemical recurrence was defined by the guidelines of NCCN. The median follow-up time of censored patients was 6.4 years. Tumor tissue samples were recognized by a pathologist through the frozen section and were snap-frozen in liquid nitrogen immediately after surgery along with paired normal tissues. The use of pathological specimens, as well as the review of all the pertinent patient record, was approved by the institutional ethics review board, and informed consent from the patients was obtained.

### Tissue microarray and immunohistochemistry

Specimens from radical prostatectomy were fixed in formalin and embedded in paraffin. A tissue core around 1 mm in diameter containing dominant tumor area was collected from each specimen and was arranged into a recipient block to form a tissue microarray. TSPAN1 antibody (Santa Cruz, sc-376551, CA, USA) was diluted (1:400) and added to the slides followed by incubation overnight at 4°C. The slides were then washed in PBS and incubated by anti-mouse EnVisionTM kit (DAKO, Glostrup, Denmark) for 30 min at 37°C. Two observers independently scored the degree of immunostaining and were clinically blind. The proportion of positively stained tumor cells was graded as: 0 (no positive tumor cells), 1 (< 10 %), 2 (10–50 %), or 3 (> 50 %). The intensity of staining was scored as 0 (no staining), 1 (weak), 2 (moderate), or 3 (strong). The staining index was calculated as the product of the two above. Specimens with staining index score ≥ 6 was considered as high expression, 3 – 4 as mid-expression, and ≤ 2 as low expression.

### Cell culture

The human prostate cancer cell lines, LNCaP and DU145 and HEK293T human embryonic kidney cells were kindly provided by the Institute of Health Sciences, Shanghai. These were cultured at 37°C in a humidified incubator (5% CO) in RPMI-1640 medium (Gibco, Carlsbad, CA, USA) supplemented with 10% fetal calf serum (FCS), 100 U/mL penicillin, and 100 μg/mL streptomycin.

### Generation of stable cell lines

Three siRNA oligos were designed according to the published TSPAN1 sequence from GenBank:

(si-1) 5′-CACCAACACAGCCAAUGAATT-3′

(si-2) 5′-GUGUCCAUGUAUCUGUACUTT-3′

(si-3′UTR) 5′- TTTAGGCGATGCCTGACTTTC-3′.

A TSPAN1 cDNA clone plasmid was purchased from Youbio, Changsha, China. The recombinant plasmids (pLKO.1-puro-shRNA TSPAN1, pCDH-puro-TSPAN1) were constructed and sequenced by Boshang Biotechnologies (Shanghai, China). LNCaP and DU145 cell lines stably expressing TSPAN1-specific shRNA or an empty vector, were constructed using the lentiviral shRNA technique. LNCaP and DU145 cells were transduced with lentiviral supernatant and selected under 2 μg/mL puromycin for 1 to 2 weeks for stable transfectants.

### RNA extraction and quantitative RT-PCR

Total RNA was isolated from cultured cells, and primary tumor samples using TRIzol reagent (Invitrogen, Carlsbad, CA, USA) and was converted into the first-strand cDNA with the first-strand cDNA synthesis kit (Tiangen Biotech Co. Ltd., Beijing, China) according to the manufacturer's instructions. Quantitative PCR was performed using SYBR Master Mix (Takara, Japan) on a LightCycler 480 System (Roche Applied Science, Basel, Switzerland). Human GAPDH gene was used as an endogenous control. Results were represented as the fold expression relative to the control. PCR primers were as follows:

human TSPAN1, forward 5′-CATGCAGTTTGTC AACGTGGG-3′ and reverse 5′-CACTTGCTCTCAG TCTTAGCAC-3;

human GAPDH, forward 5′-GACTCATGACC ACAGTCCATGC-3′ and reverse 5′-AGAGGCAGGGAT |GATGTTCTG-3′.

### Western blot analysis

Western analyzes were performed with protein lysates (Thermo, USA) obtained from cultured cells. Protein concentrations were determined using a BSA kit (Thermo, USA). 20 μg of total denatured protein was resolved on 10% or 12% SDS-polyacrylamide gels and transferred onto a nitrocellulose membrane (Millipore, Temecula, CA, USA). The membranes were blocked and probed with primary antibodies overnight at 4°C: anti-TSPAN1 (1:1000) from Abcam, Cambridge, UK; anti-AKT, anti phospho--AKT (S473), anti-ERK, anti phospho--ERK (pT202/pY204), anti-cyclinD1, anti-casepase3, and anti-cleaved caspase3 at 1:2000, respectively (Cell Signaling Technology, Danvers, MA, USA), antiβ-tubulin, anti-cyclinE, and anti-actin also at 1:2000, respectively, (Santa Cruz). Membranes were washed three times and incubated with horseradish peroxidase-conjugated secondary antibodies (Abcam). The target protein bands were visualized using enhanced chemiluminescence method exposed on X-ray films.

### Cell proliferation assay

The effects of TSPAN1 knockdown and overexpression on prostate cancer cells proliferation were determined by MTT assay. Cells were seeded into 96-well plates (5 × 10 cells/well) and cultured for 6 days. Subsequently, they were incubated with MTT (Sigma-Aldrich, St. Louis, MO, 15 μl/well) at 37°C for 4 h, followed by 200 μl DMSO addition into each well. The absorbance was measured at 570 nm.

### Cell migration assay

The degree of cell migration was assessed using the BD migration chamber (BD Biosciences, Bedford, MA), according to the manufacturer's protocol.

### Flow cytometric analysis

Replicative DNA synthesis and DNA content were analyzed using univariate flow cytometric analysis. TSPAN1 siRNA oligo transfected LNCaP cells were fixed in 70% ethanol and stored at −20°C. 2 h before flow cytometric analysis, the cells were suspended in a 1 mL modified Vindelov's DNA staining solution (10 μg/mL RNase A and 5 μg/mL propidium iodide in PBS). Cells in G0/G1, S, and G2/M phases of the cell cycle were determined with FlowJo software.

### Statistical analysis

Data were expressed as median (IQR). Survival curves were plotted by the Kaplan-Meier method and compared by the log-rank test and cox regression analysis. Statistical comparisons of the results were made using a *t-test* or non-parametric test. All tests were 2-sided, and a *p-value* < 0.05 was considered to be statistically significant. SPSS (ver. 21.0) was used to analyze all parameters.

## SUPPLEMENTARY MATERIALS FIGURE



## References

[1] Siegel RL1, Miller KD, Jemal A (2015). Cancer statistics 2015. CA Cancer J Clin.

[2] Han M, Partin AW, Zahurak M, Piantadosi S, Epstein JI, Walsh PC (2003). Biochemical (prostate specific antigen) recurrence probability following radical prostatectomy for clinically localized prostate cancer. J Urol.

[3] Briganti A, Karnes RJ, Joniau S, Boorjian SA, Cozzarini C, Gandaglia G, Hinkelbein W, Haustermans K, Tombal B, Shariat S, Sun M, Karakiewicz PI, Montorsi F (2014). Prediction of outcome following early salvage radiotherapy among patients with biochemical recurrence after radical prostatectomy. Eur Urol.

[4] Ross AE, Yousefi K, Davicioni E, Ghadessi M, Johnson MH, Sundi D, Tosoian JJ, Han M, Humphreys EB, Partin AW, Walsh PC, Trock BJ, Schaeffer EM (2016). Utility of Risk Models in Decision Making After Radical Prostatectomy: Lessons from a Natural History Cohort of Intermediate- and High-Risk Men. Eur Urol.

[5] Boorjian SA, Thompson RH, Tollefson MK, Rangel LJ, Bergstralh EJ, Blute ML, Karnes RJ (2011). Long-term risk of clinical progression after biochemical recurrence following radical prostatectomy: the impact of time from surgery to recurrence. Eur Urol.

[6] Ross AE, Johnson MH, Yousefi K, Davicioni E, Netto GJ, Marchionni L, Fedor HL, Glavaris S, Choeurng V, Buerki C, Erho N, Lam LL, Humphreys EB (2016). Tissue-based Genomics Augments Post-prostatectomy Risk Stratification in a Natural History Cohort of Intermediate- and High-Risk Men. Eur Urol.

[7] Zöller M (2009). Tetraspanins: push and pull in suppressing and promoting metastasis. Nat Rev Cancer.

[8] Todeschini AR, Dos Santos JN, Handa K, Hakomori SI (2008). Ganglioside GM2/GM3 complex affixed on silica nanospheres strongly inhibits cell motility through CD82/cMet - mediated pathway. ProcNatlAcadSci USA.

[9] Bandyopadhyay S, Zhan R, Chaudhuri A, Watabe M, Pai SK, Hirota S, Hosobe S, Tsukada T, Miura K, Takano Y, Saito K, Pauza ME, Hayashi S (2006). Interaction of KAI1 on tumor cells with DARC on vascular endothelium leads tometastasis suppression. Nat Med.

[10] Shiomi T, Inoki I, Kataoka F, Ohtsuka T, Hashimoto G, Nemori R, Okada Y (2005). Pericellular activation of proMMP - 7 (promatrilysin - 1) through interaction with CD151. Lab Invest.

[11] Huerta S, Harris DM, Jazirehi A, Bonavida B, Elashoff D, Livingston EH, Heber D (2003). Gene expression profile of metastatic colon cancer cells resistant to cisplatin induced apoptosis. Int J Oncol.

[12] Herlevsen M, Schmidt DS, Miyazaki K, Zöller M (2003). The association of the tetraspanin D6 1A with the α6β4 integrin supports cell motility and liver metastasis formation. J Cell Sci.

[13] Serru V, Dessen P, Boucheix C, Rubinstein E (2000). Sequence and expression of seven new tetraspans. Biochim Biophys Acta.

[14] Leyden J, Murray D, Moss A, Arumuguma M, Doyle E, McEntee G, O'Keane C, Doran P, Macmathuna P (2006). Net1 and Myeov: computationally identified mediators of gastric cancer. Br J Cancer.

[15] Chen L, Yuan D, Zhao R, Li H, Zhu J (2010). Suppression of TSPAN1 by RNA interference inhibits proliferation and invasion of colon cancer cells *in vitro*. Tumori.

[16] Hölters S, Anacker J, Jansen L, Beer-Grondke K, Dürst M, Rubio I (2013). Tetraspanin 1 promotes invasiveness of cervical cancer cells. Int J Oncol.

[17] Desouki MM, Liao S, Huang H, Conroy J, Nowak NJ, Shepherd L, Gaile DP, Geradts J (2011). Identification of metastasis-associated breast cancer genes using a high-resolution whole genome profiling approach. J Cancer Res ClinOncol.

[18] Sboner A, Demichelis F, Calza S, Pawitan Y, Setlur SR, Hoshida Y, Perner S, Adami HO, Fall K, Mucci LA, Kantoff PW, Stampfer M, Andersson SO (2010). Molecular sampling of prostate cancer: a dilemma for predicting disease progression. BMC Med Genomics.

[19] Troyer DA, Jamaspishvili T, Wei W, Feng Z, Good J, Hawley S, Fazli L, McKenney JK, Simko J, Hurtado-Coll A, Carroll PR, Gleave M, Lance R (2015). A multicenter study shows PTEN deletion is strongly associated with seminal vesicle involvement and extracapsular extension in localized prostate cancer. Prostate.

[20] Toren P, Zoubeidi A (2014). Targeting the PI3K/Akt pathway in prostate cancer: challenges and opportunities (review). Int J Oncol.

[21] Chen Z, Trotman LC, Shaffer D, Lin HK, Dotan ZA, Niki M, Koutcher JA, Scher HI, Ludwig T, Gerald W, Cordon-Cardo C, Pandolfi PP (2005). Crucial role of p53-dependent cellular senescence in suppression of Pten-deficient tumorigenesis. Nature.

[22] Brockman JA, Alanee S, Vickers AJ, Scardino PT, Wood DP, Kibel AS, Lin DW, Bianco FJ, Rabah DM, Klein EA, Ciezki JP, Gao T, Kattan MW (2015). Nomogram Predicting Prostate Cancer-specific Mortality for Men with Biochemical Recurrence After Radical Prostatectomy. Eur Urol.

[23] Pound CR, Partin AW, Eisenberger MA, Chan DW, Pearson JD, Walsh PC (1999). Natural history of progression after PSA elevation following radical prostatectomy. JAMA.

[24] Karnes RJ, Bergstralh EJ, Davicioni E, Ghadessi M, Buerki C, Mitra AP, Crisan A, Erho N, Vergara IA, Lam LL, Carlson R, Thompson DJ, Haddad Z (2013). Validation of a genomic classifier that predicts metastasis following radical prostatectomy in at risk patient population. J Urol.

[25] Knezevic D, Goddard AD, Natraj N, Cherbavaz DB, Clark-Langone KM, Snable J, Watson D, Falzarano SM, Magi-Galluzzi C, Klein EA, Quale C (2013). Analytical validation of the Oncotype DX prostate cancer assay – a clinical RT-PCR assay optimized for prostate needle biopsies. BMC Genomics.

[26] Cuzick J, Berney DM, Fisher G, Mesher D, Møller H, Reid JE, Perry M, Park J, Younus A, Gutin A, Foster CS, Scardino P, Lanchbury JS, Transatlantic Prostate Group (2012). Prognostic value of cell cycle progression signature for prostate cancer death on conservatively managed needle biopsy cohort. Br J Cancer.

[27] Edlind MP, Hsieh AC (2014). PI3K-AKT-mTOR signaling in prostate cancer progression and androgen deprivation therapy resistance. Asian J Androl.

[28] Barbieri CE, Rubin MA (2015). Genomic rearrangements in prostate cancer. CurrOpin Urol.

[29] Ahearn TU, Pettersson A, Ebot EM, Gerke T, Graff RE, Morais CL, Hicks JL, Wilson KM, Rider JR, Sesso HD, Fiorentino M, Flavin R, Finn S (2015). A Prospective Investigation of PTEN Loss and ERG Expression in Lethal Prostate Cancer. J Natl Cancer Inst.

[30] Mithal P, Allott E, Gerber L, Reid J, Welbourn W, Tikishvili E, Park J, Younus A, Sangale Z, Lanchbury JS, Stone S, Freedland SJ (2014). PTEN loss in biopsy tissue predicts poor clinical outcomes in prostate cancer. Int J Urol.

