# Supplementary material for: Decreased TSPAN1 promotes prostate cancer progression and is a marker for early biochemical recurrence after radical prostatectomy
Source: Oncotarget. 2016 Aug 20;7(39):63294–305. doi: 10.18632/oncotarget.11448 (PMC5325364; doi:10.18632/oncotarget.11448)
Supplement: Supplementary file 1 [file oncotarget-07-63294-s001.pdf]

Decreased TSPAN1 promotes prostate cancer progression and is a marker for early biochemical recurrence after radical prostatectomy

Supplementary Materials

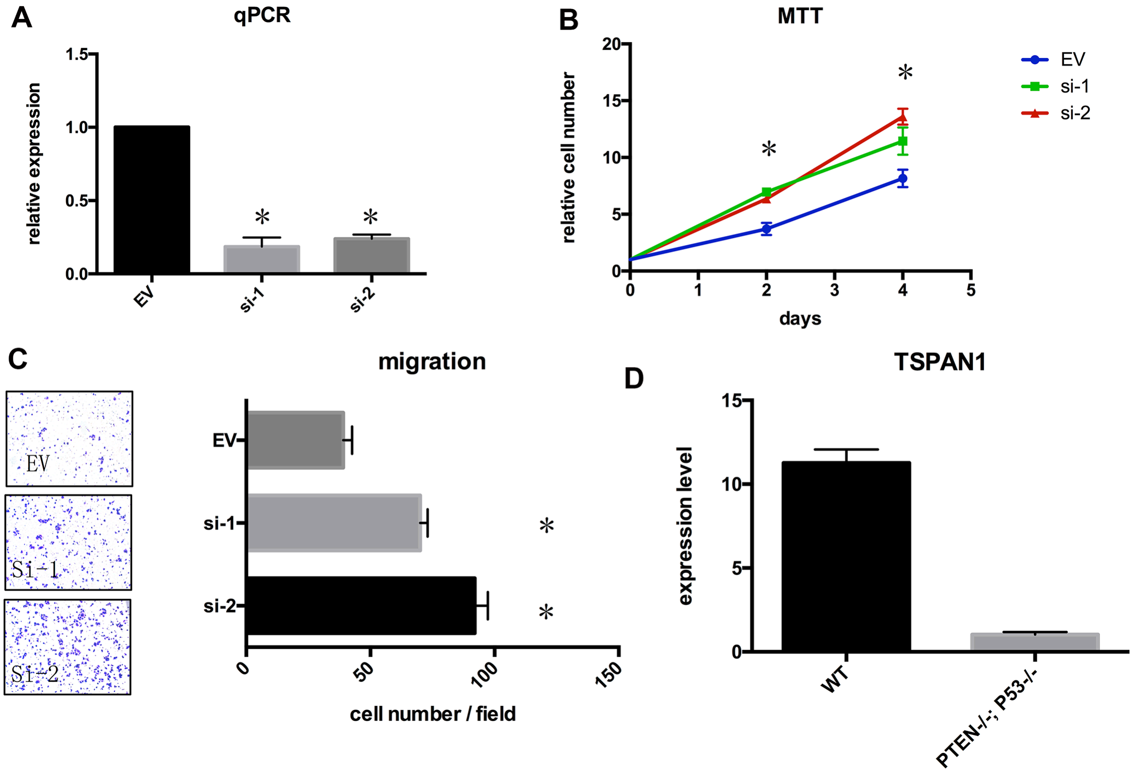

Supplementary Figure S1: (A) TSPAN1 knockdown in PC3 cells was checked by qPCR test. (**B**) MTT essay of TSPAN1 knockdown in PC3 cells. (**C**) Migration essay of TSPAN1 knockdown in PC3 cells. (**D**) qPCR analysis of TSPAN1 expression in WT and PTEN−/−; P53−/− mouse.
